# Differential effects of alprazolam and clonazepam on the immune system and blood vessels of non-stressed and stressed adult male albino rats

**DOI:** 10.2478/v10102-011-0021-y

**Published:** 2011-09

**Authors:** Ghada E. Elmesallamy, Marwa A. Abass, Nahla A.G. Ahmed Refat, Amal H. Atta

**Affiliations:** 1 Department of Forensic Medicine and Clinical Toxicology, Faculty of Medicine, Zagazig University, Egypt; 2 Department of Pathology, Faculty of Veterinary Medicine, Zagazig University, Egypt; 3 Department of Microbiology, Faculty of Medicine, Zagazig University, Egypt

**Keywords:** alprazolam, clonazepam, stress, SRBC, IL-2

## Abstract

Benzodiazepines belongs to one of the most commonly used anxiolytic and anticonvulsant drugs in the world. Full description of toxic effects on different organs is lacking for nearly all the current benzodiazepines. The aim of the current work was to study the immunologic and vascular changes induced by sub-chronic administration of alprazolam and clonazepam in non-stressed and stressed adult male albino rats. Forty-two adult male albino rats were divided into 6 groups (I): (Ia) Negative control rats, (Ib): Positive control rats received distilled water, (II): Stressed rats, (III): Non-stressed rats received daily oral dose of clonazepam (0.5 mg/kg), (IV): Stressed rats received daily oral dose of clonazepam (0.5 mg/kg), (V): Non-stressed rats received daily oral dose of alprazolam (0.3 mg/kg). (VI): Stressed rats received daily oral dose of alprazolam (0.3 mg/kg). At the end of the 4th week, total leukocyte count (WBCs) and differential count were determined, anti-sheep RBC antibody (Anti-SRBC) titer and interleukin-2 (IL-2) level were assessed, thymus glands, lymph nodes, spleens and abdominal aortae were submitted to histopathological examination. Alprazolam was found to induce a significant increase in neutrophil count and a significant decrease in lymphocytes, anti-SRBC titer and IL-2 level with severe depletion of the splenic, thymal and nodal lymphocytes, accompanied by congestion and eosinophilic vasculitis of all organs tested in comparison to clonazepam treated rats. Stress enhanced the toxic effects. It was concluded that the immune system and blood vessels can be adversely affected to a greater extent by short-term chronic administration of alprazolam than by clonazepam, and these toxic effects are aggravated by stress.

## Introduction

Recent studies have found that stress plays a role in the etiology of many diseases. Stress is generally considered to be immunosuppressive and to increase susceptibility to infections and cancer. Paradoxically, it also exacerbates inflammatory and autoimmune diseases. Although it is well established that stress alters the release of various hormones and neurotransmitters, the mechanisms by which stress affects immune responses remain elusive (Viswanathan *et al.,* 2005; Salak-Johnson & McGlone, 2007).

Benzodiazepines (BZD) are among the most commonly used groups of anxiolytic drugs in the world. They are indicated for treatment of generalized anxiety disorders, treatment of panic disorders with or without agoraphobia, sedation, light anesthesia and anterograde amnesia of perioperative events, control of seizures, and skeletal muscle relaxation (Iqbal *et al.,* 2002).

Clonazepam, a benzodiazepine derivative, is used for the treatment of epilepsy, psychiatric and neurologic disorders, including panic disorders. It has also been utilized in alleviating movement disorders and restless leg syndrome in patients with end-stage renal disease (Brouns & De Deyn 2004; Morishita, 2009).

Alprazolam is one of the most commonly prescribed short-acting benzodiazepines in the childbearing period. It has replaced diazepam in drug prescription for treatment of anxiety disorders. This shift in the pattern of drug prescription was justified by the low likelihood of its accumulation (compared with diazepam) and by the sedative effects of multiple doses (Pinna *et al.,* 1997).

All benzodiazepines, including clonazepam and alprazolam, act by enhancing γ-aminobutyric acid GABA-ergic neurotransmission through the binding on specific BZD recognition sites, within the GABA (A) receptor-ion channel complex. However, it has been found that BZD also act on peripheral benzodiazepine receptor sites (PBR) or translocator protein 18kDa (TSPO) (Gavish *et al.,* 1999). Evidence for a direct immunomodulatory action for BZD emerged from recent studies demonstrating the presence of TSPO on immune/inflammatory cells (De Lima *et al.,* 2010).

The production of antigen-specific antibodies represents a major defense mechanism of humoral immune responses. Data suggest that the primary antibody response to anti-sheep RBC antibody (Anti-SRBC) may be one of the most sensitive endpoints available to assess chemically induced alterations of the immune system. This endpoint has become the cornerstone of several recently established guidelines for assessing the potential immunotoxicity of xenobiotics (Ladics, 2007).

Interleukin 2 (IL-2) is secreted primarily by activated T-lymphocytes and natural killer (NK) cells. It is necessary for regulatory T cell maturation in the thymus and may also sensitize antigen-activated T cells to apoptosis. The importance of IL-2 in supporting the immune response makes this cytokine and its receptor a prime target for both activation and suppression. Additionally, its inhibition is critical for immunosuppression (Malek, 2003; Leo & Hsieh, 2008).

On reviewing the available literature from a toxicological point of view it was noticed that there was little information regarding the influence of clonazepam and alprazolam on different body tissues and cells, including the immune and vascular system. Most of the studies were focused on their alteration of the immune function rather than the structure. Therefore it was thought to be of particular interest to study the toxic effects of these commonly used drugs on these tissues at their maximum therapeutic doses.

The aim of the current work was to study the differential immunologic and vascular changes induced by sub-chronic administration of clonazepam and alprazolam in non stressed and stressed adult male albino rats.

## Material and methods

### Drugs

Clonazepam was obtained from Roche, F. Hoffmann-La Roche LTd, Basel, Switzerland in the form of a sterile oral solution containing 2.5 mg/ml, supplied with its dropper (1 drop contains 0.1 mg clonazepam). On administration, each 0.5 mg of the drug was diluted with 5 ml of distilled water.

Alprazolam in the form of white crystalline powder, was obtained from Amoun Pharmaceutical Industries Co., freshly prepared for oral gavage by dissolving it in distilled water (each 0.3 mg dissolved in 5 ml of distilled water).

### Kits

Mouse Interleukin-2 (IL-2) ELISA kit of Bioscience, Inc, Catalog Number: 88-7024 was used as an enzyme-linked immunosorbent assay for quantitative measurement of IL-2 in serum.

### Sheep RBCs

Sheep RBCs were obtained from the laboratory of Zoology Department, Faculty of Veterinary Medicine, Zagazig University.

### Experimental design

Forty-two adult male albino rats of 200 g average weight were brought from the Animal House, Faculty of Medicine, Zagazig University, divided into 6 groups and caged under standardized environmental conditions.

- **Group I:** Twelve rats were equally subdivided into **(Ia):** Negative control non-stressed rats, received only the regular diet and tap water to determine the normal values of the performed tests. **(Ib):** Positive control non-stressed rats, gavaged daily with 1 ml of distilled water.
- **Group II:** Six stressed rats, were submitted to daily restraint stress at room temperature.
- **Group III:** Six non stressed rats were given daily oral dose of Clonazepam (0.5 mg/kg) (Paget & Barnes, 1964) for 4 weeks.
- **Group IV:** Six stressed rats were given daily oral dose of Clonazepam (0.5 mg/kg) for 4 weeks.
- **Group V:** Six non-stressed rats were given daily oral dose of Alprazolam (0.3 mg/kg) (Paget & Barnes, 1964) for 4 weeks.
- **Group VI:** Six stressed rats were given daily oral dose of Alprazolam (0.3 mg/kg) for 4 weeks.

Both drugs were assessed in their maximum therapeutic dose used for treatment of generalized anxiety disorders.

At the end of the study, the rats were anesthetized with ether and 5 ml blood was collected from their retro-orbital plexus. Total leukocyte count and differential count were determined and immunological studies were performed by measuring the anti-sheep RBC hemagglutination (anti-SRBC) titer and the level of serum Interleukin-2 (IL-2). The rats were then sacrificed, the thymus glands, lymph nodes, spleens and abdominal aortae were dissected and submitted to histopathological examination.

### Restraint stress procedure

It was done by the procedure described by Glavin *et al.* (1994). Stress was applied by placing the animals (without squeezing or compression) in well-ventilated wire mesh restrainers for a single daily session of 2.5 h beginning at 9:00 a.m. This procedure mimics stress that is largely psychological in nature.

### WBCs count and differential count

Total leukocyte count was determined by following the method described by Chanarin *et al.* (1973), using Coulter T660 hematology analyzer, Beckman Coulter, Inc., USA. Two ml of blood were collected in tubes containing 20 µl EDTA solution and differential count was done on Leishman stained peripheral blood smear.

### Humoral immune response

It was assayed by the Sheep RBC Antibody Titer (SRBC): In this test 1.5 ml of blood was collected, the serum was separated and analyzed for hemagglutination titer according to the method described by Ladics *et al.* (2000). Two-fold dilutions (0.025 mg) of sera were made in the microtiter plates with saline. To each well 0.025 ml of 1% (v/v/) SRBC was added. The plates were incubated at 37 °C for 1 h and then observed for hemagglutination. The highest dilution giving hemagglutination was taken as the antibody titer. The antibody titers were expressed in a graded manner, the minimum dilution (1/2) being ranked as 1. The mean ranks of different groups were compared for statistical analysis.

### Cell-mediated immune response

It was assayed through determination of the cytokine Interleukin-2 (IL-2): 1.5 ml blood samples were left to clott and were then centrifuged at 3 000 rpm for 10 minutes. Serum samples were separated and kept frozen at −20 °C for determination of interleukin-2 (IL-2) by Enzyme Linked-Immuno-Sorbent Assay (ELISA) according to the method described by Hollander *et al.* (1998). The data analysis program automatically determined the concentrations of IL-2 in the samples by comparing the absorbency of the samples to the standard curve which demonstrated an inverse relationship between Optical Density (O.D.) and the cytokine concentration. IL-2 concentration was represented as pg/ml.

### Histopathological examination

Paraffin blocks and sections of thymus glands, lymph nodes, spleens and abdominal aortae were prepared, stained with hematoxylin and eosin and subjected to routine histological examination by the method described by Bancroft & Stevens (1996).

### Statistical analysis

SPSS Software program was used. Mean values (M)±SD were calculated. t-test and F test were performed. P value of less than 0.05 was considered to be significant.

## Results

At the end of the 4^th^ week of the current study, comparison between negative control rats (Group Ia) and positive control rats (Group Ib) showed a non-significant difference in all parameters tested (*p>*0.05) (Table 1)

**Table 1:** Immunologic parameters in the different study groups after 4 weeks (Anova test).

		Controls								
Test		Group IA	Group IB	Group II	Group III	Group IV	Group V	Group VI	F	*p-*value
Total Leukocyte Count a		8.41±1.10	8.43±0.75	8.39±0.82	8.37±1. 30	8.31±0.61	8.28±0.61	8.21±0.7	0.05	0.90
Differential count a	Lymphocytes	7.21±0.7	6.8±1.9	6.7±1.4	6.8±1.7	6.6±1.3	6.5±1.4	5.1±0.5	2.39	0.03*
	Neutrophils	0.90±0.3	0.9±0.6	1.8±0.3	1.0±0.2	1.9±0.8	1.0±0.5	1.8±0.2	5.98	0.001***
	Eosinophils	0.8±0.1	0.8±0.1	0.8±0.09	0.8±0.1	0.8±0.1	0.8±0.0	0.8±0.1	0.31	0.92
	Monocytes	0.41±0.1	0.4±0.3	0.4±0.3	0.4±0.2	0.4±0.2	0.4±0.2	0.4±0.3	0.04	0.99
Anti-SRBC titre		5.87±0.32	5.95±0.33	4.50±0.25	4.00±0.32	3.27±0.25	4.77±0.32	3.18±0.22	83.4	0.001***
IL-2 (pg/ml)		38.0±6.2	38.1±5.5	36.7±6.7	35.5±3.1	32.6±6.7	33.0±5.2	28.4±6.1	11.78	<0.01**

Group IA=negative control; Group IB=positive control; Group II=stressed only (no drug); Group III=clonazepam only (no stress);

Group IV = clonazepam+stress; Group V=alprazolam only (no stress); Group VI=alprazolam+stress

a All values shown are Mean (±SD) in terms of (×10^3^/mm^3^); n=6 per group.

* Significant: p<0.05

** p<0.01

*** p<0.001

Stressed non-treated rats (Group II) showed a significant increase (*p<*0.05) in the mean values of neutrophils as compared to control group. However, they showed a non-significant change regarding the mean values of WBCs, lymphocytes, eosinophils and monocytes, when compared with those of the control group (*p>*0.05). They also exhibited a significant decrease (*p<*0.001) in the mean values of anti-SRBC titer and a non-significant decrease (*p>*0.05) in the mean values of IL-2 compared to the control group, as shown in Table 1.

**Figure 1:**
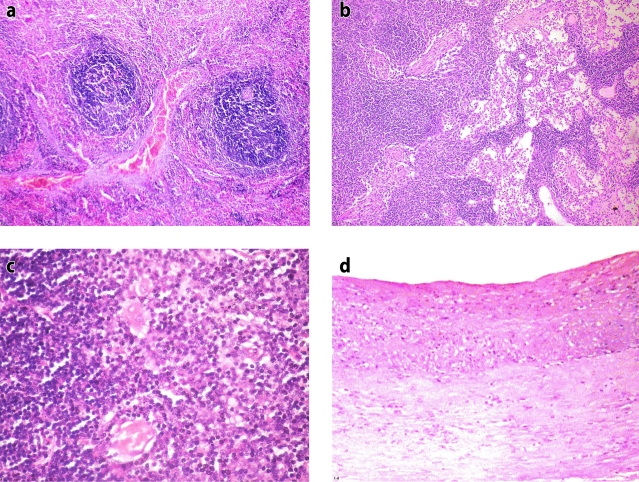
Control rat (group I): a) Spleen section showing normal structure (H&E, × 200), b) Lymph node section showing normal structure (H&E, × 200), c) Thymus section showing normal structure (H&E, × 400), d) Large artery section showing normal structure (H&E, × 200).

**Figure 2:**
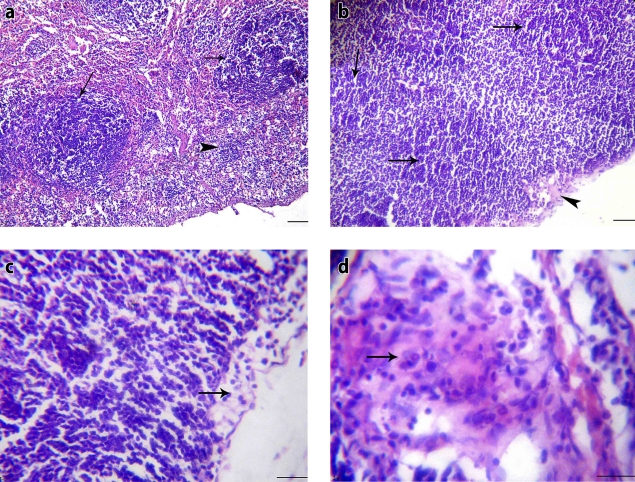
Stressed non-treated rat (group II): a) Spleen section showing normal white pulp and proliferation of reticular cells (H&E, × 200), b) Lymph node section showing normal lymphoid follicles and mild subcapsular edema (H&E, × 200), c) Thymus section showing mild subcapsular edema (H&E, × 400), d) Large artery section showing eosinophilic vasculitis and eosinophils infiltrations (H&E, × 400).

Histopathological examination of the same group (Group II) showed minimal changes in the spleens and lymph nodes in the form of slight subcapsular edema, as seen in Figures (2-a, 2-b), when compared to the normal architecture of the control group (Figures 1-a, 1-b). The thymuses showed a mild subcapsular edema, while the aortae displayed eosinophilic vasculitis (Figures 2-c, 2-d) as compared to the control group (Figures 1-c, 1-d).

Clonazepam-treated non-stressed rats (Group III) showed a non-significant change (*p>*0.05) in the mean values of WBCs, lymphocytes, neutrophils, eosinophils and monocytes when compared with those of the control group (Group I). They also exhibited a significant decrease (*p<*0.001) in the mean values of anti-SRBC titer and a non significant decrease (*p>*0.05) in the mean value of IL-2 level as compared to the control group. (Table 1).

Comparison between the clonazepam-treated non stressed rats (Group III) and the stressed non-treated rats (Group II) showed a non-significant change (*p>*0.05) in the mean values of all immunologic parameters tested except neutrophils. A significant increase (*p<*0.05) in the mean values of neutrophils was observed when compared with Group II, as shown in Table 2.

**Table 2:** Comparison of the mean values for each parameter assessed in the different study groups (LSD test).

	WBC	Lymphocytes	Neutrophils	Eosinophils	Monocytes	anti*-*SRBC	IL-2
a*p*_1__(I__vs.__II)_	0.972	0.727	0.01	0.904	0.909	0.001***	0.390
*p*_2__(I__vs.__III)_	0.950	0.773	0.684	0.945	0.965	0.001***	0.064
*p*_3__(I__vs.__IV)_	0.854	0.663	0.037*	0.828	0.895	0.001***	0.001***
*p*_4__(I__vs.__V)_	0.811	0.432	0.768	0.873	0.929	0.001***	0.001***
*p*_5__(I__vs.__VI)_	0.720	0.047*	0.007**	0.781	0.997	0.001***	0.001***
*p*_6__(II__vs.__III)_	0.973	0.934	0.019*	0.949	0.892	0.127	0.364
*p*_7__(II__vs.__IV)_	0.872	0.932	0.801	0.947	0.997	0.001***	0.016*
*p*_8__(II__vs.__V)_	0.824	0.777	0.053	0.994	0.865	0.396	0.0198*
*p*_9__(II__vs.__VI)_	0.725	0.189	0.919	0.898	0.926	0.001***	0.001***
*p*_10__(III__vs.__IV)_	0.917	0.863	0.051	0.873	0.879	0.032*	0.0395*
*p*_11__(III__vs.__V)_	0.876	0.683	0.977	0.928	0.969	0.033*	0.0518
*p*_12__(III__vs.__VI)_	0.786	0.1302	0.008**	0.819	0.973	0.016*	0.001***
*p*_13__(IV__vs.__V)_	0.948	0.859	0.079	0.9217	0.849	0.001***	0.780
*p*_14__(IV__vs.__VI)_	0.834	0.236	0.847	0.936	0.919	0.752	0.0123*
*p*_15__(V__vs.__VI)_	0.883	0.226	0.034*	0.856	0.946	0.001***	0.001***

Group IA=negative control; Group IB=positive control; Group II=stressed only (no drug); Group III=clonazepam only (no stress); Group IV=clonazepam+stress;

Group V=alprazolam only (no stress); Group VI=alprazolam+stress.

a Groups being compared shown in parentheses.

* Significant: *p<*0.05

** *p<*0.01

*** *p<*0.001

**Figure 3:**
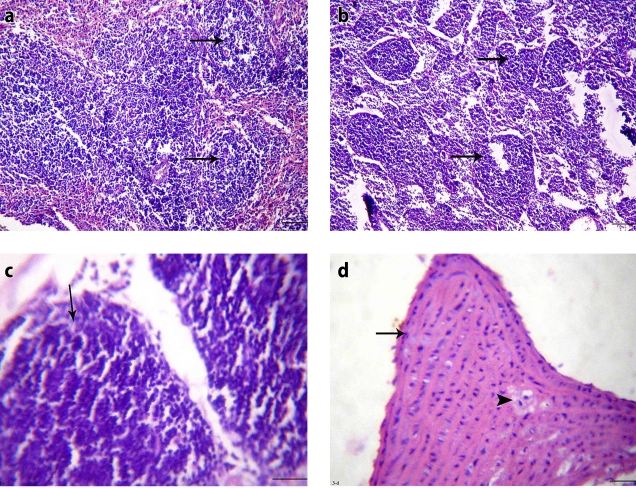
Clonazepam-treated unstressed rat (group III): a) Spleen section showing normal white pulp and congested red pulp (H&E, × 200), b) Lymph node section showing mild edema among slightly depleted lymphoid follicles (H&E, × 200), c) Thymus section showing normal subcapsular lymphoid follicles (H&E, × 200), d) Large artery section showing plaque protruded inside the arterial lumen and presented by proliferation of smooth muscles and deposition of some foam cells (H&E, × 200).

The observed changes in the tested organs of the same group (Group III) were in the form of congested red pulp of the spleens with normal white pulp and slight depletion of the lymphoid follicles in the lymph node specimens (Figures 3-a, 3-b). Thymus glands showed subcapsular lymphoid follicles (Figure 3-c). Examination of the aortic tissues revealed multiple plaques protruding inside the arterial lumen. These plaques were composed of smooth muscles and deposition of some foam cells (Figure 3-d).

Clonazepam-treated stressed rats (Group IV) revealed a significant increase (*p<*0.05) in the mean values of neutrophils. However, the mean values of WBCs, lymphocytes, eosinophils, and monocytes showed a non-significant change (*p>*0.05) when compared with control rats (Group I). There was also a significant decrease (*p<*0.001) in the mean values of anti-SRBC titer and IL-2 level as compared to the control group (Tables 1 and 2)

**Figure 4:**
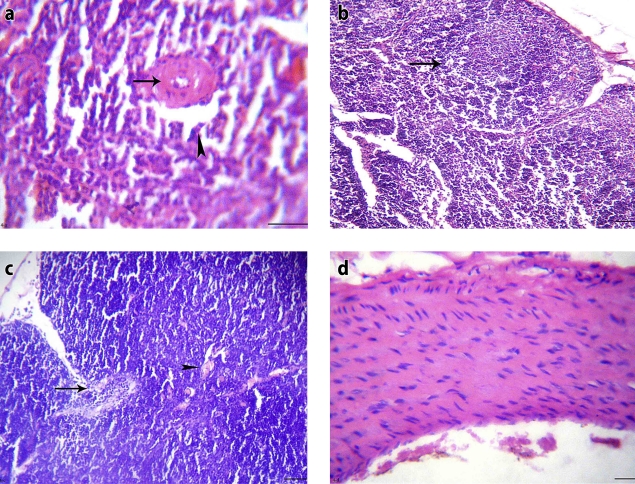
Clonazepam-treated stressed rat (group IV): a) Spleen section showing hyaline degeneration of the wall of splenic arteriole and perivascular edema (H&E, × 400), b) Lymph node section showing mild depletion of lymphocytes from subcapsular lymphoid follicles (H&E, × 400), c) Thymus section showing mild depletion of the cortical lymphoid follicles and medullary congestion (H&E, × 200), d) Large artery section showing normal media and intima (H&E, × 200).

Moreover, in stressed rats (Group IV) clonazepam treatment was found to induce hyaline degeneration of the wall of splenic arterioles with perivascular edema (Figure 4-a). Lymph nodes examination showed mild depletion of lymphocytes from subcapsular lymphoid follicles (Figure 4-b). The thymuses exhibited mild depletion of the cortical lymphoid follicles and medullary congestion (Figure 4-c). However, the media and intima of the arterial wall presented no changes (Figure 4-d).

Alprazolam-treated non-stressed rats (Group V) showed a non-significant change (*p>*0.05) in the mean values of WBCs, lymphocytes, neutrophils, eosinophils and monocytes when compared with control rats (Group I). The mean values of anti-SRBC titer and IL-2 level presented a significant decrease (*p<*0.001) as compared to the control group (Tables 1 and 2)

**Figure 5:**
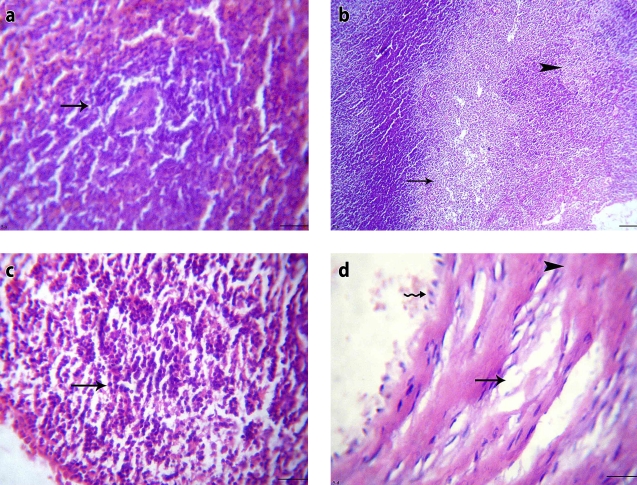
Alprazolam-treated unstressed rat (group V): a) Spleen section showing depletion of the marginal lymphocytes in the white pulp (H&E, × 400), b) Lymph node section showing mild depletion of lymphocytes from subcapsular lymphoid follicles (H&E, × 200), c) Thymus section showing depletion of lymphocytes from lymphoid follicles and mild interstitial edema (H&E, × 200), d) Large artery longitudinal section showing wide space (edema) between degenerated muscles and endotheliosis (H&E, × 400).

Spleen sections of the same group (Group V) showed depletion of the marginal lymphocytes in the white pulp (Figure 5-a) and lymph nodes had a slight depletion of the lymphoid follicles, as shown in Figure 5-b. Thymus glands also had a mild depletion of lymphocytes from the lymphoid follicles and mild interstitial edema (Figure 5-c). On examination of aortic sections, edematous walls, composed of degenerated muscles with endotheliosis were observed (Figure 5-d).

Comparison between alprazolam-treated stressed rats (Group VI) and control rats (Group I), showed a significant decrease (*p<*0.05) in the mean values of lymphocytes and a significant increase (*p<*0.05) in those of neutrophils. In contrast, a non-significant change (*p>*0.05) was detected in the mean values of WBCs, eosinophils and monocytes. The mean values of anti-SRBC and IL-2 presented a significant decrease (*p<*0.001) when compared with those of control rats. (Tables 1 and 2).

On comparing the immunologic findings of alprazolam-treated stressed rats (Group VI) with those of the stressed non-treated rats (Group II), a significant decrease (*p<*0.001) was seen in the mean values of anti-SRBC titer and IL-2 level (Table 2)

**Figure 6:**
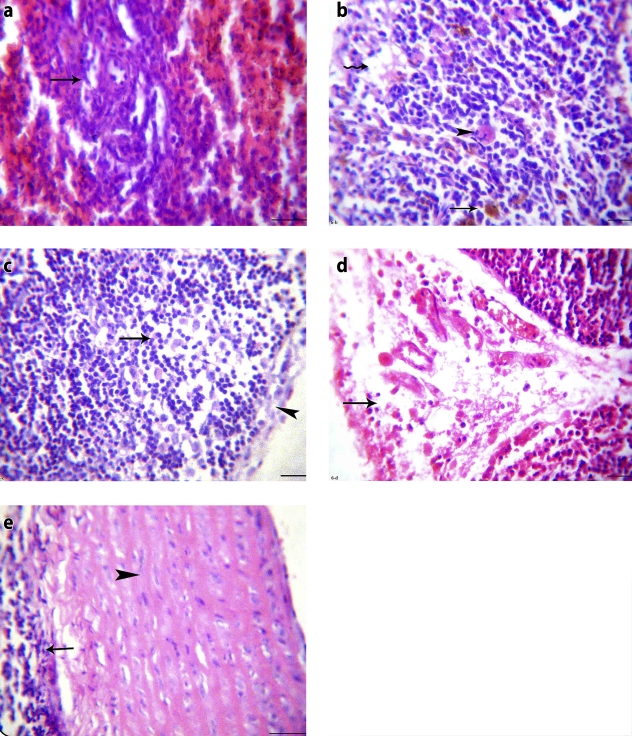
Alprazolam-treated stressed rat (group VI): a) Spleen section showing severe depletion of lymphocytes from the white pulp and extensive hemorrhages in the red pulp (H&E, × 400), b) Spleen section showing hemosiderosis, degenerated megakaryocytes and edema in the red pulp besides histiocyte infiltrations(H&E, × 400), c) Lymph node section showing focal replacement of subcapsular follicles with reticular cells and macrophages (H&E, × 400), d) Thymus section showing subcapsular edema, congested capillaries and few erythrocyte and leukocyte infiltrations (H&E, × 200), e) Large artery section showing eosinophilic vasculitis with thick tunica media and eosinophil and round cell infiltrations in the tunica adventitia (H&E, × 200).

Histopathological findings of the same group (Group VI) supported the previous results. Severe depletion of the white pulp lymphocytes and extensive hemorrhages in the red pulp with hemosiderosis and degenerated megakaryocytes were noticed on examination of spleen sections. Moreover, edema and histocyte infiltrations were also observed in the red pulp of the spleens (Figures 6-a, 6-b). Focal replacement of subcapsular follicles of the lymph nodes by reticular cells and macrophages was also detected (Figure 6-c). Furthermore, thymus glands showed marked edema and congestion with few erythrocyte and leukocyte infiltrations (Figure 6-d). Eosinophilic vasculitis was evident on examination of aortic sections with thick tunica media. Eosinophils and round cell infiltrations in the tunica adventitia were also noticed, as shown in Figure 6-e.

When comparing clonazepam-treated non-stressed rats (Group III) and alprazolam-treated non-stressed rats (Group V), a non-significant difference (*p>*0.05) was found in most of the parameters tested. An exception was the anti-SRBC titer, as alprazolam induced a significant decrease (*p<*0.05) in its mean values when compared to those of clonazepam-treated non-stressed rats. Comparison between clonazepam-treated stressed rats (Group IV) and alprazolam-treated stressed rats (Group VI), showed that alprazolam induced a significant decrease (*p<*0.05) in the mean values of IL-2 level compared with those induced by clonazepam (Table 2)

Finally, on observing the differential toxic effects of clonazepam treatment in non-stressed rats (Group III) and in stressed rats (Group IV), a significant decrease (*p<*0.05) in the mean values of anti-SRBC titer and IL-2 level was found in the clonazepam-treated stressed rats. Regarding the differential toxic effects of alprazolam treatment in non-stressed rats (Group V) and stressed rats (Group VI), it was found that alprazolam induced a significant increase (*p<*0.05) in the mean values of neutrophils and a significant decrease in the mean values of anti-SRBC titer and IL-2 level in stressed rats (Group VI) compared to non-stressed rats (Group V), as shown in Table 2.

## Discussion

Benzodiazepines (BZPs) are widely used drugs as tranquilizers, anticonvulsants and in various other indications as light anesthesia and skeletal muscle relaxation. However, not all benzodiazepines have been tested for their immunotoxic effects (Huemer *et al.,* 2010).

In the current study two commonly prescribed BZPs (clonazepam and alprazolam) were selected to study their immunologic and vascular toxic potential. Both anti-SRBC titer and IL-2 level were used to assess the humoral and cell-mediated immune functions.

IL-2 is necessary for the maturation of thymus lymphocytes, facilitation of immunoglobulin production made by B cells, and the differentiation and proliferation of natural killer cells (Malek, 2003; Waldmann, 2006).

Moreover, it was observed that small numbers of sheep red blood cells (SRBC) markedly augmented the proliferation of T lymphocytes activated by antigens or mitogens. This effect occurred with as few as one SRBC per T lymphocyte and with intact or osmotically lysed red cells. It was also accompanied by increase in interleukin-2 (Guo & White, 2010).

After 4 weeks of clonazepam and alprazolam administration, more pronounced immunologic and vascular toxic effects were demonstrated in stressed than in non-stressed adult male albino rats. These toxic changes were significantly expressed in the immunologic parameters as neutrophils, lymphocytes, anti-SRBC, and IL-2 (especially in alprazolam-treated stressed rats), supported by the histopathological findings as depletion of lymphocytes from the spleens, lymph nodes and thymuses, congestion, edema, hemosiderosis, inflammatory cells infiltration, large vessels eosinophilic infiltration, vasculitis, plaques and degeneration.

Our results are in accordance with those of Massoco and Palermo-Neto (2003) and Huemer *et al.* (2010) who stated that many benzodiazepines induced prolonged impairment of cellular immune functions in experimental animals after chronic low-dosage administration.

It has been substantiated that stressful stimuli in man as well as in animals lead to suppression of the humoral and cellular components of the immune system. The central nervous system is known to be involved in the regulation of stress-induced immune responsiveness (Yin *et al.,* 2000).

The results of the present study also showed that restraint stress produced an inhibition of SRBC antibody, that was worsened by the administration of either clonazepzm or alprazolam with inhibition of IL-2. Kalashnikov *et al.* (2002) found that exposure to low doses of BDZ resulted in long-lasting reduction of TNF-alpha, IL-1, IL-6, IL-2 and interferon-gamma.

In 1991, Chang *et al.* found that alprazolam induced severe inhibitory effects on the proliferative responses of both B- and T-cells. It also reduced the production of IL-2 by splenic T-cells, IL-1 and tumor necrosis factor (TNF) by peritoneal macrophages. Moreover, long-lasting depression of lymphocytic proliferation has been described in offspring of rats exposed to either diazepam or clonazepam during pregnancy (Schlumpf *et al.,* 1991).

Regarding the toxic effects on differential WBC counts, Bautista-Quach *et al.* (2010) reported a case of pancytopenia following oral administration of 0.25 mg clonazepam twice a day for approximately two weeks.

Lymphocytic depletion, similar to that observed in the current study, was noticed in both red and white pulp of the spleen in rats treated with diazepam (0.1 mg) daily for 4 weeks (Moris, 1991). Moreover, the observed hemosideriosis in spleens of alprazolam-treated stressed rats may be attributed to increased erythrophagia (Pacheco and Santos, 2002). In this context, it was found that diazepam accelerateed RBCs destruction via inhibition of Ca^+2^ ATPase on RBCs membrane (Seckin *et al.,* 2007).

Several reports identified BZP peripheral type binding sites (PBR) in endocrine steroidogenic tissues, organs and cells of the immune system, such as macrophages and lymphocytes. Thus, the PBR may be a possible primary target for the immunotoxic effects of BDZ (Righi *et al.,* 1999).

In the current study, immune and vascular toxic effects might be related to cortisol production. West *et al.,* (2001) stated that stimulation of PBR in steroidogenic tissues such as the adrenals increaseed glucocorticoid production. Glucocorticoid hormones are known for their potent immunosuppressive and anti-inflammatory properties.

Corticosteroids have been shown to promote the immune response during acute stress and to inhibit the immune response during chronic stress (Dhabhar & McEwen, 1997). On the other hand, restraint stress was found to induce corticosterone secretion (Li *et al.,* 2000).

Elevated endogenous corticosteroid levels were linked to reduced spleen cellularity and B cell function in mice (Shi *et al.,* 2003). Moreover, they produced neutrophilic leukocytosis, lymphopenia and reduced anti-SRBC titer and IL-2 level, a picture similar to that detected in the present study (Anderson *et al.,* 1999; Obmiñska-Mrukowicz & Szczypka, 2004).

Alprazolam was tested previously for its effects on corticosteroid production. Chronic administration of alprazolam for 29 days to hamster rats resulted in increased cortisol and total glucocorticoid levels. It was concluded that alprazolam had a stimulative effect on cortisol production (Arvat *et al.,* 1999).

On the other hand, clonazepam was shown to counteract the effect of stress on cortisol level (Chevassus *et al.*, 2004).

In this context, the cytokine system emerges as a good candidate. Indeed, the production and release of cytokines are known to mediate both inflammatory and immune responses (Wiegers & Reul, 1998) and not only cortisol (Almawi *et al.,* 1996). Schlumpf *et al.,* (1994) reported that PBR stimulation of macrophage and lymphocyte membranes changed the cytokine network.

Interestingly, the action of the PBR ligands seems to be connected with blockage of voltage-dependant Ca^+2^ channels (Ostuni *et al.,* 2004). Calcium release appears to be essential for T cell activation, cytokine synthesis, and proliferation. Both increases and decreases in intracellular Ca^2+^ have been linked to apoptosis (Lepple-Wienhues *et al.,* 1999; Mason, 1999).

Clonazepam was found to have a strong binding capacity to peripheral benzodiazepine receptors in rat aortic smooth muscles compared to other benzodiazepines. These binding sites were concentrated in the mitochondria (Cox *et al.,* 1991).

Peripheral benzodiazepine receptors bind with high affinity to cholesterol and transport it across the mitochondrial membrane (Papadopoulos *et al.*, 1997). This may explain the appearance of foam cells and degeneration of smooth muscle observed in the aortae of clonazepam treated rats.

Tanimoto *et al.* (1999) found that stimulation of the thymus PBR induced apoptosis in thymocytes. This action was accentuated by dexamethasone administration.

Ekonomopoulou *et al.,* (2010) reported that alprazolam, diazepam, and lorazepam exhibited cytogenetic activity in normal human lymphocyte cultures. A possible mutagenic action could explain their immunotoxic effects (Giri & Banerjee, 1996).

Furthermore, an *in vitro* study carried out by Saha *et al.* (2009) concluded that alprazolam strongly interacted with DNA, resulting in conformational changes in the DNA. Similarly, Iakovidou-Kritsi *et al.,* (2009) found that alprazolam induced genotoxicity and cytotoxicity in human lymphocyte cell culture at doses equivalent to oral doses, with a significant increase of Sister Chromatid Exchanges on peripheral human lymphocytes *in vivo*.

It seems relevant to point out that alprazolam has a triazolo- ring, and a – CH_3_ – group that could interact with DNA as an alkylating agent (Brambilla *et al.,* 2007).

In contrast to the present results, Freire-Garabal *et al.,* (1991) found that chronic administration of alprazolam for one month alleviated stress-induced suppression of thymus and spleen cellularity in mice after laparotomy. Covelli *et al.,* (1998) also reported that alprazolam behaved as an immunoenhancer (enhancing the function.of immune cells).

Clonazepam and alprazolam appeared to modulate immune responsiveness in both non-stressed and stressed animals, albeit in a different manner, and these effects were mediated via alteration of the structure of organs of the immune system (histopathological lesions).

It was concluded that the immune system and blood vessels can be adversely affected by sub-chronic administration of alprazolam to a greater extent than by clonazepam, and these toxic effects are aggravated by stress. Further, the results of the present study raise concern regarding the safety of benzodiazepine administration over long periods.

## References

[CIT0001] Almawi WY, Beyhum HN, Rahme AA, Rieder MJ (1996). Regulation of cytokine and cytokine receptor expression by glucocorticoids. J Leukocyte Biol.

[CIT0002] Anderson BH, Watson DL, Colditz IG (1999). The effect of dexamethasone on some immunological parameters in cattle. Vet Res Commun.

[CIT0003] Arvat EB, Maccagno J, Ramunni L, Di Vito R, Giordano L, Gianotti F, Broglio F, Camanni M, Ghigo E (1999). The inhibitory effect of alprazolam, a benzodiazepine, overrides the stimulatory effect of metyrapone-induced lack of negative cortisol feedback on corticotrophin secretion in humans. J Clin Endocrinol Metabol.

[CIT0004] Bancroft CD, Stevens A (1996). Theory and practice of histological techniques. 4th edition.

[CIT0005] Bautista-Quach MA, Liao YM, Hsue CT (2010). Pancytopenia associated with clonazepam. J Hematol Oncol.

[CIT0006] Brambilla G, Carrozzino R, Martelli A (2007). Genotoxicity and carcinogenicity studies of benzodiazepines. Pharmacol Res.

[CIT0007] Brouns R, De-Deyn PP (2004). Neurological complications in renal failure: a review. Clin Neurol Neurosurg.

[CIT0008] Chanarin I, Cairns J, Waters D (1973). Coulter blood count. J Clin Pathol.

[CIT0009] Chang MP, Castle SC, Norman DC (1991). Suppressive effects of alprazolam on the immune response of mice. Int J Immunopharmacol.

[CIT0010] Chevassus H, Mourand I, Molinier N, Lacarelle B, Jean-Frédéric B, Petit P (2004). Assessment of single-dose benzodiazepines on insulin secretion, insulin sensitivity and glucose effectiveness in healthy volunteers: a double-blind, placebo-controlled, randomized cross-over trial. BMC Clin Pharmacol.

[CIT0011] Covelli V, Maffione AB, Nacci C, Tatò E, Jirillo E (1998). Stress, neuropsychiatric disorders and immunological effects exerted by benzodiazepines. Immunopharmacol Immunotoxicol.

[CIT0012] Cox DA, Ellinor PT, Kirley TL, Matlib MA (1991). Identification of a 17-kDa protein associated with the peripheral-type benzodiazepine receptor in vascular and other smooth muscle types. J Pharmacol Exp Ther.

[CIT0013] De Lima CB, Sakai M, Latorre AO, Moreau RL, Palermo-Neto J (2010). Effects of different doses and schedules of diazepam treatment on lymphocyte parameters in rats. Int Immunopharmacol.

[CIT0014] Dhabhar FS, McEwen BS (1997). Acute stress enhances while chronic stress suppresses cell- mediated immunity in vivo: a potential role for leukocyte trafficking. Brain Behav Immun.

[CIT0015] Ekonomopoulou MT, Tsoleridis CA, Argyraki M, Polatoglou E, Stephanidou-Stephanatou J, Iakovidou-Kritsi Z (2010). Cytogenetic activity of newly synthesized 1,5-benzodiazepines in normal human lymphocyte cultures. Genet Test Mol Biomarkers.

[CIT0016] Freire-Garabal M, Belmonte A, Orallo F, Couceiro J, Núñez MJ (1991). Effects of alprazolam on T-cell immunosuppressive response to surgical stress in mice. Cancer Letter.

[CIT0017] Gavish M, Bachman I, Shoukrun R, Katz Y, Ve enman L, Weisinger G, Weizman A (1999). Enigma of the peripheral benzodiazepine receptor. Pharmacol Rev.

[CIT0018] Giri AK, Banerjee S (1996). Genetic toxicology of four commonly used benzodiazepines: A review. Mut Res Rev Gen Tox.

[CIT0019] Glavin GB, Paré WP, Sandbak T, Bakke HK, Murison R (1994). Restraint stress in biomedical research: an update. Neuroscie Biobehav Rev.

[CIT0020] Guo TL, White KL (2010). Methods to assess immunotoxicity. Comprehensive Toxicology, 2^nd^ ed.

[CIT0021] Hollander GA, Zuklys S, Morel C, Mizoguchi E, Mobisson K, Simpson S, Terhorst C, Wishart W, Golan DE, Burakoff SJ (1998). Monoallelic expression of the interleukin-2 locus. Scie.

[CIT0022] Huemer HP, Lassnig C, Nowotny N, Irschick EU, Kitchen M, Pavlic M (2010). Diazepam leads to enhanced severity of orthopoxvirus infection and immune suppression. Vaccine.

[CIT0023] Iakovidou-Kritsi Z, Akritopoulou K, Ekonomopoulou MT, Mourelatos D (2009). In vitro genotoxicity of two widely used benzodiazepines: alprazolam and lorazepam. Aristotle Univ Med J.

[CIT0024] Iqbal MM, Sobhan T, Ryals T (2002). Effects of commonly used benzodiazepines on the fetus, the neonate, and the nursing infant. Psychiatr Serv.

[CIT0025] Kalashnikov SV, Kalashnikova EA, Kokarovtseva SN (2002). Immunomodulating effects of tofizopam (Grandaxin) and diazepam in vitro. Mediators Inflamm.

[CIT0026] Ladics GS (2007). Use of SRBC antibody responses for immunotoxicity testing. Animal Models in Immunotoxicol.

[CIT0027] Ladics GS, Smith C, Bunn TL, Dietert RR, Anderson PK, Wiescinski CM, Holsapple MP (2000). Characterization of an approach to developmental immunotoxicology assessment in the rat using SRBC as the antigen. Toxicol Methods.

[CIT0028] Leo CW, Hsieh CS (2008). Antigen-specific peripheral shaping of the natural regulatory T cell population. J Exp Med.

[CIT0029] Lepple-Wienhues A, Belka C, Laun T, Jekle A, Walter B, Welz M, Heil L, Kun J, Weller M, Gulbins E, Lang F (1999). Stimulation of CD95 (Fas) blocks T lymphocyte calcium channels through sphingomy. Proc Natl Acad Sci USA.

[CIT0030] Li K, Liege S, Moze SE, Neveu PJ (2000). Plasma corticosterone and immune reactivity in restrained female C3H mice. Stress.

[CIT0031] Malek TR (2003). The main function of IL-2 is to promote the development of T regulatory cells. J Leuko Biol.

[CIT0032] Mason RP (1999). Calcium channel blockers, apoptosis and cancer: is there a biologic relationship?. J Am Coll Cardiol.

[CIT0033] Massoco C, Palermo-Neto J (2003). Effects of midazolam on equine innate immune response: a flow cytometric study. Vet Immunol Immunopathol.

[CIT0034] Moris GA (1991). Autoradiographic studies on the effect of diazepam on the proliferation of rat spleen lymphocytes. Egy Ger Soc Zool.

[CIT0035] Morishita S (2009). Clonazepam as a therapeutic adjunct to improve the management of depression: a brief review. Hum. Psychopharmacol.

[CIT0036] Obmiñska-Mrukowicz B, Szczypka M (2004). Effects of lysozyme dimer on the cellular and humoral response in hydrocortisone treated mice. Pol J Food Nutr Sci.

[CIT0037] Ostuni MA, Marazova K, Peranzi G, Vidic B, Papadopoulos V, Ducroc R, Lacapere JJ (2004). Functional characterization and expression of PBR in rat gastric mucosa: stimulation of chloride secretion by PBR ligands. Am J Physiol Gastrointest.

[CIT0038] Pacheco M, Santos MA (2002). Biotransformation, genotoxic, and histopathological effects of environmental contaminants in European eel (Anguilla L.). Ecotoxicol Environ Safety.

[CIT0039] Paget GE, Barnes JM, Laurence DR, Bacharach AL (1964). Toxicity tests. Evaluation of drug activities pharmacometrics.

[CIT0040] Papadopoulos V, Amri H, Boujrad N, Cascio C, Culty M, Garnier M, Hardwick M, Li H, Brown AS, Reversa JL, Drieu K (1997). Peripheral benzodiazepine receptor in cholesterol transport and steroidogenesis. Steroids.

[CIT0041] Pinna G, Galici R, Schneider HH, Stephens DN, Turski L (1997). Alprazolam dependence prevented by substituting with the (-carboline abecarnil. Proc Natl Acad Sci.

[CIT0042] Righi DA, Pinheiro SR, Guerra JL, Palermo-Neto J (1999). Effects of diazepam on Mycobacterium bovis-induced infection in hamsters. Braz J Med Biol Res.

[CIT0043] Saha B, Mukherjee A, Santra CR, Chattopadhyay A, Ghosh AN, Choudhuri U, Karmakar P (2009). Alprazolam intercalates into DNA. J Biomol Struct Dyn.

[CIT0044] Salak-Johnson JL, McGlone JJ (2007). Making sense of apparently conflicting data: stress and immunity in swine and cattle. J Anim Sci.

[CIT0045] Schlumpf M, Ramseier H, Lichtensteiger W (1991). Prenatal diazepam induced persisting depression of cellular immune responses. Life Scie.

[CIT0046] Schlumpf M, Lichtensteiger W, Van Loveren H (1994). Impaired host resistance to Trichinella spiralis as a consequence of prenatal treatment of rats with diazepam. Toxicol.

[CIT0047] Seçkin S, Alsancak S, Ba(aran-Küçükgergin C, Uy M (2007). The effect of chronic diazepam administration on lipid peroxidation and Ca 2+ -ATPase activity in rat liver. Acta Biol Hung.

[CIT0048] Shi Y, Devadas S, Greeneltch KM, Yin D, Mufson RA, Zhou JN (2003). Stressed to death: Implication of lymphocyte apoptosis for psycho neuroimmunology. Br Beh Imm.

[CIT0049] Tanimoto Y, Onishi Y, Sato Y, Kizaki H (1999). Benzodiazepine receptor agonist modulate thymocyte apoptosis through reduction of the mitochondrial transmembrane potential. Jpn J Pharmacol.

[CIT0050] Viswanathan K, Daugherty C, Dhabhar FS (2005). Stress as an endogenous adjuvant: augmentation of the immunization phase of cell-mediated immunity. Int Immunol.

[CIT0051] Waldmann TA (2006). The biology of interleukin-2 and interleukin-15: implications for cancer therapy and vaccine design. Nature Rev Immun.

[CIT0052] West LA, Horvat RD, Roess DA, Barisas BG, Juengel JL, Niswender GD (2001). Steroidogenic acute regulatory protein and peripheral-type benzodiazepine receptor associate at the mitochondrial membrane. Endocrinol.

[CIT0053] Wiegers GJ, Reul JM (1998). Induction of cytokine receptors by glucocorticoids: Functional and pathological significance. Trends in Pharmacol Scie.

[CIT0054] Yin D, Tuthill RD, Mufson A, Shi Y (2000). Chronic restraint stress promotes lymphocyte apoptosis by modulating Cd95 expression. J Exp Med.

